# The specificity of CTBP dehydrogenase inhibitors MTOB and 4-Cl-HIPP

**DOI:** 10.17912/micropub.biology.001415

**Published:** 2025-02-03

**Authors:** Benjamin A. Stickland, Franziska Greulich, Nina Henriette Uhlenhaut

**Affiliations:** 1 Metabolic Programming, TUM School of Life Sciences, Technical University of Munich, Munich, Bavaria, Germany; 2 ZIEL-Institute for Food & Health, Technical University of Munich, Munich, Bavaria, Germany; 3 Institute for Diabetes and Endocrinology (IDE), Helmholtz Zentrum München, Munich, Bavaria, Germany; 4 German Center for Diabetes Research, Munich, Bavaria, Germany

## Abstract

C-terminal binding proteins (CTBPs) are conserved transcriptional repressors important in cancer and inflammation. Uniquely amongst transcriptional co-regulators, CTBPs possess a functional dehydrogenase domain. Since multiple malignancies display elevated CTBP levels, CTBP inhibitors targeting this dehydrogenase domain have been developed. While the importance of CTBPs dehydrogenase function for transcriptional regulation remains unclear, several studies have relied on CTBP inhibitors. *In vitro* experiments have confirmed binding of these compounds to CTBP’s active site, however evidence for specificity is lacking. To address this, we treated wildtype and *Ctbp1* , *2* double knockout J774.1 cells with MTOB or 4-Cl-HIPP and performed RNA-seq. We observed that both inhibitors elicit distinct transcriptional changes indicating non-overlapping modes of action. Moreover, the majority of changes induced by either inhibitor are observed in *Ctbp1/2* double knockout cells suggesting off-target effects. We hypothesize that those CTBP dehydrogenase inhibitors lack specificity to CTBPs and emphasise careful revaluation of findings inferred from studies using those inhibitors.

**Figure 1. Off-targets of CTBP inhibitors MTOB and 4-Cl-HIPP in J774.1 cells:**
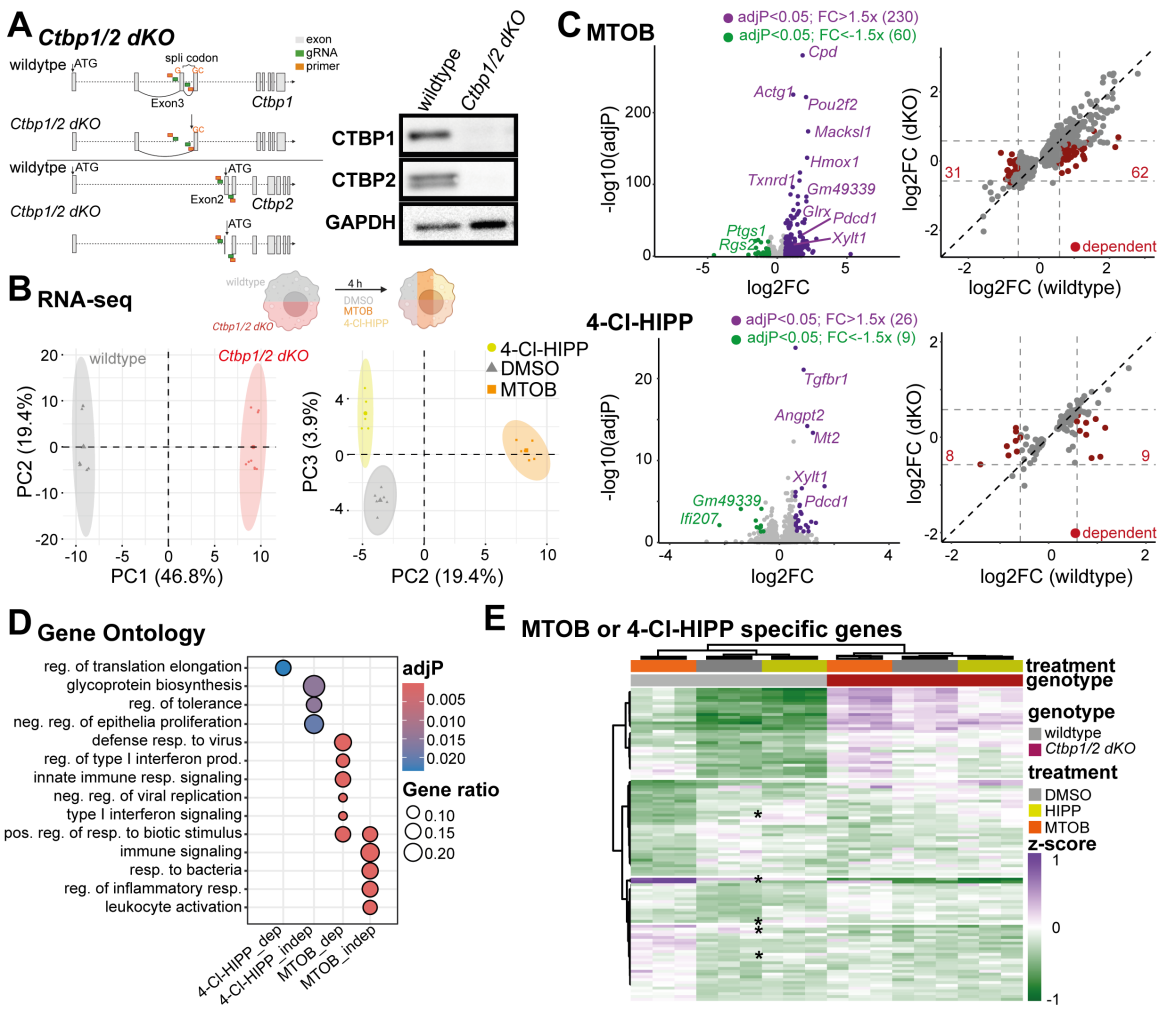
**(A)** Overview of the CRISPR double knockout ( *dKO* ) strategy and confirmation of CTBP1 and CTBP2 protein loss by western blot. We have introduced a premature STOP and frameshift mutation in both, the *Ctbp1* and *2* gene. **(B)** Principal component analysis from RNA-seq data of wildtype (grey) and *Ctbp1/2 dKO* (red) J774.1 cells after PBS/DMSO (control, grey), 4-Cl-HIPP (250 µM, yellow) and MTOB (1.5 mM, orange) treatment. N=3 **(C)** Differential expression of MTOB (top) or 4-Cl-HIPP (bottom) treated J774.1 wildtype cells. Significantly induced genes (Benjamini-Hochberg-adjusted P-value (adjP) < 0.05, Fold change (FC) > 1.5-fold) are indicated in purple, repressed genes (adjP < 0.05, FC > 1.5-fold) in green. Selected genes are labelled. Plots on the right show the correlation of fold changes observed in wildtype (x-axis) or *Ctbp1/2 dKO* (dKO, y-axis) cells after treatment with the respective inhibitor. Genes with significant changes in wildtype (adjP < 0.05), that do not respond to the respective inhibitor in the *Ctbp1/2 dKO* are termed CTBP-dependent (red). Numbers represent the number of genes. N=3 **(D)** Overrepresentation analysis of genes with CTBP-dependent (dep) or independent (indep) inhibitor effect by gene ontology for biological process. Colors indicate Benjamini-Hochberg-adjusted P-values, dot size the gene ratio. neg. – negative, reg. – regulation, resp. – response **(E)** Heatmap of genes that are CTBP1/2-dependently regulated by MTOB or 4-Cl-HIPP. Colors show the z-scaled variance-stabilized read counts. Clustering was performed by Euclidian distance. * indicate selected 4-Cl-HIPP regulated genes interblended with the MTOB target genes. &nbsp;

## Description

Here we report a RNA-seq study that elucidates the specificity of two substrate competitive inhibitors of C-terminal binding proteins (CTBP) 1 and 2.

CTBPs are paralogous transcriptional co-regulators that harbour a d-isomer specific 2-hydroxyacid dehydrogenase domain (D2-HDH) and their overexpression is associated with cancer (Boyd, Subramanian et al., 1993, Dcona, Morris et al., 2017, Schaeper, Boyd et al., 1995). Additionally, CTBPs are mediators of inflammation (Li, Zhang et al., 2020, Li, Zhang et al., 2022, Saijo, Collier et al., 2011, Shen, Kapfhamer et al., 2017). Therefore, inhibition of CTBP function may provide clinical tools to target malignancies or chronic inflammatory diseases.

The D2-HDH domain of CTBPs reduces or oxidises yet unknown substrates using the co-enzyme NAD+/NADH. 4-methylthio-2-oxobutanoate (MTOB) as a substrate analogue that binds the substrate-binding pocket of CTBPs in a CTBP-specific way (Hilbert, Grossman et al., 2014) , thereby antagonizing transcriptional regulation by CTBPs (Straza, Paliwal et al., 2010) . Using MTOB as a lead structure, 1 ^st^ generation CTBP inhibitors such 2-(hydroxyimino)-3-phenylpropanoic acid and its 2- and 4-chloro analogues were developed (Korwar, Morris et al., 2016) .

To test the specificity of the two CTBP inhibitors MTOB and 4-Cl-HIPP (3-(4-Chlorophenyl)-2-(hydroxyimino) propanoic acid), we generated a *Ctbp1/2* double knockout (dKO) in the macrophage cell line J774.1 ( **Fig. 1A** ). Wildtype and *Ctbp1/2 dKO* cells were treated with either DMSO as vehicle control, 1.5 mM MTOB or 250 µM 4-Cl-HIPP for 4 h to investigate direct drug effects on transcription. The concentrations of both inhibitors were chosen in accordance with published dose response curves (Achouri, Nöel et al., 2007, Dcona, Chougoni et al., 2023, Dcona, Damle et al., 2019, Hilbert, Morris et al., 2015, Straza, Paliwal et al., 2010). RNA was collected and drug effects assessed by RNA-seq.

Principal component analysis of the RNA-seq results shows a clear separation of double knockout and wildtype cells, explaining 46.8% of variance. Of the inhibitors, MTOB has the strongest influence on gene expression, driving 19.4% of gene expression variation. 4-Cl-HIPP, on the other hand effects only 3.9% of variation. Surprisingly, the inhibitor effects were observed in wildtype and *Ctbp1/2 dKO* cells on this global view, indicating off-target effects of both inhibitors ( **Fig. 1B** ).

A more detailed analysis of gene expression in response to either MTOB or 4-Cl-HIPP revealed 290 and 35 differentially expressed genes in wildtype cells, respectively ( **Fig. 1C** ). A comparison of inhibitor effects in wildtype to the effect in *Ctbp1/2 dKO* cells showed that both inhibitors also effect gene expression even when CTBPs 1 and 2 are not expressed further confirming their off-target effects. For MTOB, 93 of the 290 regulated genes were CTBP-dependent ( **Fig. 1C** top right), whereas for 4-Cl-HIPP 17 of the 35 genes showed CTBP dependency ( **Fig. 1C** right bottom). Among the CTBP-dependent 4-Cl-HIPP effected genes “regulation of translation elongation” enriched significantly by overrepresentation analysis for gene ontologies of biological process. MTOB, on the other hand, regulates “defense response to viruses”, “innate immune response signaling” and “type I interferon signaling” ( **Fig. 1D** ). Those results indicate that 4-Cl-HIPP and MTOB do regulate different gene sets, even among their CTBP-dependent target genes hinting towards a different mode of action. To further elucidate the similarities or differences of both inhibitors, we specifically looked at CTBP-dependently regulated genes for both inhibitors (17 for 4-Cl-HIPP, 93 for MTOB) by blotting the z-scaled variance stabilized counts as heatmap and performed clustering analysis by Euclidian distance. Again, MTOB had the stronger effect of the two inhibitors as it contributed 93 genes to the gene set. Among the CTBP-dependent MTOB target genes we identified genes mimicking the *Ctbp1/2* double knockout effect and genes that do not mimic the effect of *Ctbp1/2* loss-of-function ( **Fig. 1E** ). The CTBP-dependent MTOB effects that are not mimicked by *Ctbp1/2* loss-of-function may depend on its dehydrogenase activity or oligomerization state.

In summary, the two CTBP inhibitors MTOB and 4-Cl-HIPP have distinct modes of actions of which only a subset is mimicked by *Ctbp1/2* loss-of-function. More importantly, both inhibitors show effects in *Ctbp1/2* double knockout cells indicative for off-target effects. We could not identify specific pathways enriched among the off-target genes, but other D2-HDH-containing dehydrogenases may potentially be targeted by those inhibitors. One potential candidate is phosphoglycerate dehydrogenase (PHGDH). Its loss was recently associated with an M2 to M1 phenotype switch in tumour-associated macrophages (Cai, Li et al., 2024) .

## Methods

*Generation of Ctbp1 and 2 double knockout cells*

To assemble a functional gRNA, 2 µl Alt-R CRISPR-Cas9 tracrRNA labelled with ATTO 550 and 2 µl crRNA (IDT, 100 µM) were mixed in 20 µl duplex buffer, heated to 95°C for 5 min and slowly cooled back to room temperature within two hours. 0.7 µl annealed oligonucleotides (50 µM) were mixed with 0.5 µl Alt-R Cas9 (IDT) in 1.8 µl PBS and incubated for 20 min at room temperature to assemble RNP complexes. 500,000 cells of the cell line J774.1 were washed with PBS and resuspended in 12 µl electroporation buffer R (Thermo Fisher Scientific). 4 µl of electroporation enhancer (15 µM IDT), 8 µl of electroporation buffer R and 12 µl cell suspension were added to the assembled RNP complexes. The solution was mixed with a 10 µl electroporation tip, the filled tip was placed in the Neon electroporation device (Thermo Fisher Scientific) in 5 mL buffer E and subjected to 3x 1400 V for 10 ms each. Then cells were seeded in 6-well plates pre-filled with full growth medium. Next day, cells were suspended in FACS buffer (5 mM EDTA, 2% FBS, PBS), pipetted through a cell strainer to obtain single cell suspension and subjected to FACS (BD Aria II). To enrich for successfully transfected cells ATTO 550 positive cells were sorted in a container filled with growth medium. Cells were diluted to 2 cells/ 100 µl and 100 µl aliquots were seeded on 96-well plates for single cell outgrowth. During this time, cells were monitored closely to ensure monoclonal origin. After reaching confluence, clonal colonies were propagated and subjected to genotyping by PCR. Selected clones were confirmed as either wildtype or knockout by sequencing of PCR products and western blotting.

*Inhibitor treatment of J774.1*

J774.1 wildtype or *Ctbp1/2* double knockout cells were seeded at 250,000 cells/ well of a 24-well plate in 1 mL full growth medium (DMEM high glucose, 10 % FBS, 1 % Penicillin/ Streptomycin) and incubated at 37 °C and 5 % CO _2_ overnight. Next day, cells were treated with 1.5 mM MTOB (2 M in PBS, Sigma Aldrich Cat. No.: K6000), 250 µM 4-Cl-HIPP (500 mM in DMSO, gifted by W.E.Royer) or a vehicle control (PBS/ DMSO) for 4 h.

*Western blot*

Cells were lysed in RIPA buffer (150 mM NaCl, 50 mM Tris (pH 7.4), 1% Nonidet P-40, 0.5% sodium-deoxycholate, 0.1% sodium-dodecylsulfate). 6x Laemmli-buffer (375 mM Tris-HCl (pH 6.8), 6% SDS, 4.8 % glycerol, 9% 2-mercaptoethanol and 0.03% bromphenolblue) was added to a final concentration of 1X and protein lysates were boiled at for 10 min 95 °C. Proteins were separated by size using a 8% polyacrylamide gel in running buffer (25 mM Tris, 192 mM glycine, 0.1 % SDS) and transferred to a PVDF membrane in transfer buffer (25 mM Tris, 192 mM glycine, 20 % ethanol) using semi-dry transfer. Membranes containing protein lysates were blocked for 1 h in 5 % BSA in TBS-T (150 mM NaCl, 10 mM Tris , 0.1 % Tween-20) and incubated with primary antibody overnight at 4°C. The primary antibody was removed and membranes were washed thrice with TBS-T before incubation with HRP-coupled secondary antibodies for 1 h at room temperature. Membranes were washed again thrice with TBS-T before HRP substrate was added. Chemiluminescence was detected using the Sapphire Azure Bioscanner.

*RNA-seq*

Total RNA was extracted from treated cells using the ReliaPrep RNA Miniprep System (Promega Cat. No.:Z6012) according to the manufacturer’s instructions. The RNA quality was determined on an Agilent 2100 Bioanalyzer with the RNA 6000 Nano kit, following the manufacturer’s instructions. Library preparation and rRNA depletion were conducted using the TruSeq unstranded mRNA Library Prep kit (Illumina) starting with 1 µg of RNA for each biological triplicate. The samples were sequenced on the Illumina NovaSeq6000 with 2x100bp paired-end.

*Data analysis*

NGS data quality was assessed with FastQC (RRID: SCR 014583, http://www.bioinformatics.babraham.ac.uk/projects/fastqc/).

Gene-level quantification was performed with Salmon version 1.9.0 (RRID: SCR_017036 (Patro, Duggal et al., 2017) ). Settings were: -libType A, -gcBias, -biasSpeedSamp 5 using the mm39 (M28, GRCm39) reference transcriptome provided by Gencode (Frankish, Diekhans et al., 2021) . Gene count normalization and differential expression analysis were performed with DESeq2 version 1.44.0 (RRID: SCR_015687 (Love, Huber et al., 2014) ) after import of gene-level estimates with “tximport” version 1.32.0 (RRID: SCR_016752 (Soneson, Love et al., 2015) ) in R (RRID: SCR_001905, R version 4.1.1 (Team, 2018) ).

For gene annotation, Ensembl gene IDs were mapped to symbols using the Bioconductor package “AnnotationHub” version 3.12.0 (RRID: SCR_024227 (Morgan & Shepherd, 2024) ). Genes with expression higher than a 10 ^th^ percentile, fold change of 1.5, and Benjamini-Hochberg-adjusted p value < 0.05 were called significantly changed. Plots were generated with “ggplot2” version 3.5.1 (RRID: SCR_014601 (Wickham, 2016) ) or “pheatmap” version 1.0.12 (RRID: SCR_016418; https://github.com/raivokolde/pheatmap ) packages and GO enrichment performed with “clusterProfiler” version 4.12.6 (RRID: SCR 016884 (Yu, Wang et al., 2012) ).

*Data availability*

RNA-seq data is available at GSE281668.

For interactive data exploration: https://franzig.shinyapps.io/ctbpinhibitors/.

## Reagents

**Table:** 

**Target**	**Sequence**	**Manufacturer**
*Ctbp1*	AAGCCCGUUUCCAUUUACCA GUUUUAGAGCUAUGCU	IDT
*Ctbp1*	UGAGCGGCCAGUCAAACCAG GUUUUAGAGCUAUGCU	IDT
*Ctbp2*	GGGGCCGUUCAUGAUCUGG GUUUUAGAGCUAUGCU	IDT
*Ctbp2*	GUUGCACCUCACCUUUACCU GUUUUAGAGCUAUGCU	IDT

Table 1. CrRNA for CRISPR clones

**Table:** 

**Target**	**Cat. No.**	**LOT**	**RRID**	**Manufacturer**
CTBP1	612042	1209713	AB_399429	BD Biosciences
CTBP2	612044	1327035	AB_399431	BD Biosciences
Vinculin	13901	7	AB_2728768	Cell Signaling
anti-mouse IgG (HRP-coupled)	7076		AB_330924	Cell Signaling
anti-rabbit IgG (HRP-coupled)	111-035-003		AB_2313567	Jackson ImmunoResearch Labs

Table 2. Antibodies for Western blot

**Table:** 

**Name**	**Sequence**
CTBP1_geno_F	CCCCATTGTTTTGGGCATGG
CTBP1_geno_R	ACACGCTGATGCAGAGTCAA
CTBP2_geno_F	AAGGCTGTGTCCCGTCCTG
CTBP2_geno_R	AGCCACTACCGATTCGCACG

Table 3. Primer sequences

**Table:** 

**Software/package**	**Version**	**RRID**	**Reference**
FastQC		SCR 014583	http://www.bioinformatics.babraham.ac.uk/projects/fastqc/
Salmon	1.9.0	SCR_017036	(Patro et al., 2017)
R	4.4.1	SCR_001905	(Team, 2018)
DESeq2	1.44.0	SCR_015687	(Love et al., 2014)
tximport	1.32.0	SCR_016752	(Soneson et al., 2015)
AnnotationHub	3.12.0	SCR_024227	(Morgan & Shepherd, 2024)
ggplot2	3.5.1	SCR_014601	(Wickham, 2016)
pheatmap	1.0.12	SCR_016418	https://github.com/raivokolde/pheatmap
clusterProfiler	4.12.6	SCR 016884	(Yu et al., 2012)

Table 4. Software
